# Development and Validation of a Patient Satisfaction Questionnaire for Home-Based Radiography Services

**DOI:** 10.7759/cureus.98253

**Published:** 2025-12-01

**Authors:** Panagiotis Bidikoudis, Konstantinos Athanasakis, Christos Michail, Georgios Dounias

**Affiliations:** 1 Department of Public Health Policy, University of West Attica, Athens, GRC; 2 Department of Radiology, General Hospital of Nikaia-Piraeus, Piraeus, GRC; 3 Department of Biomedical Engineering, University of West Attica, Athens, GRC

**Keywords:** mobile radiography units, patient satisfaction, quality of healthcare services, questionnaire, validation

## Abstract

Introduction: The increasing demand for home-based radiography services has highlighted the need for reliable tools to assess patient satisfaction. This study aimed to develop and psychometrically validate a patient-reported questionnaire to evaluate satisfaction with home-based radiography in Greece.

Methods: The questionnaire was developed through a literature review and expert consensus using two Delphi rounds, with items retained based on ≥80% agreement and a content validity ratio of >0.78. A cross-sectional validation study was conducted with 78 participants. Construct validity was assessed through exploratory factor analysis (EFA) with varimax rotation, and internal consistency was evaluated using Cronbach’s alpha.

Results: EFA identified a two-factor model that explained 57.8% of the total variance. Cronbach’s alpha values were 0.74 and 0.71 for the two factors, respectively. The Kaiser-Meyer-Olkin measure was 0.62, and Bartlett’s test of sphericity was significant (p < 0.01). These findings indicate acceptable construct validity and internal consistency for the proposed model.

Conclusions: The developed questionnaire demonstrated satisfactory psychometric properties, supporting its use in assessing patient satisfaction with home-based radiography services. Future studies should employ confirmatory factor analysis with larger and more diverse populations, as well as cross-cultural validation, to further strengthen the instrument’s applicability and generalizability.

## Introduction

Population aging and the expansion of telemedicine have increased demand for radiographic examinations conducted outside hospital settings. Studies have shown that the use of the mobile X-ray unit can prove beneficial for patients whose mobility is limited, providing quality and safe care, while at the same time, its economic impact has been shown to be favorable through cost-benefit studies [1-5]. More specifically, several advantages of this healthcare service provision are highlighted, such as the improvement of mental health [6], the reduction of patient waiting times, the decrease in hospitalizations and readmissions, and the reduction in the transmission of new infections.

Over the past decade, there has been a steadily growing global interest in the study and research of patient satisfaction, along with the simultaneous evaluation of the quality of healthcare services. All stakeholders involved in the development of health strategies and policies are concerned both with the study of patient satisfaction as an outcome of the healthcare provided in relation to its cost and with the impact that satisfaction has on access to and use of healthcare services. Home-based radiography enhances accessibility for elderly or immobile patients but requires tools to ensure service quality and patient satisfaction.

In recent years, due to the fiscal crisis, Greek users of healthcare services have been reported, according to Eurobarometer, as dissatisfied with the provision of services they receive compared to the residents of other European Union member states [7]. Although several studies have been conducted on the quality of healthcare services, none of them have examined patient satisfaction with home-based radiological examinations. Existing satisfaction instruments, such as the Patient Satisfaction Questionnaire (PSQ)-18 and SERVQUAL, assess general healthcare services but fail to capture specific aspects of home-based radiography, including patient-technologist interaction and the logistical context of in-home examinations.

Therefore, this study aimed to develop and psychometrically validate a patient-reported questionnaire to assess satisfaction with home-based radiography services.

## Materials and methods

The present questionnaire was developed and validated using a standard scientific approach (see the Appendix). First, a systematic literature review was conducted on patient satisfaction in mobile or home radiography, resulting in a pool of 12 relevant items. Themes consistently appearing in at least three studies were retained, achieving content saturation. The topics identified were as follows: 1) the overall level of satisfaction with the home X-ray service, 2) satisfaction with the examination process, 3) satisfaction with the communication of examination results, and 4) possible psychological effects on the patient after the X-ray.

Subsequently, a Delphi study was carried out involving nine experts with experience in home healthcare services [8]: four radiologic technologists, four nurses, and one health visitor. The nine experts participated in two Delphi rounds. For each individual question, the panel was asked to evaluate whether the item being measured was “essential,” “useful but not essential,” or “not necessary,” and a content validity ratio (CVR) was calculated. Depending on the number of experts participating, the minimum acceptable CVR should not fall below a certain threshold. In our case, items with a CVR greater than 0.78 were included in the questionnaire distributed to participants [9]. The final questionnaire consisted of eight questions, while four items were excluded.

A pilot study with 20 participants was then conducted to test face validity. The participants assessed the relevance and clarity of the questions. Minor linguistic adjustments were made based on feedback. Following adjustments to reduce ambiguity, the final version of the questionnaire was produced.

The final cross-sectional study included 78 adults from the general population of Attica who used the home X-ray service during the period from March 2025 to April 2025. Thus, a convenience sample of a sufficient number of participants was obtained. Trained, nonaffiliated technologists collected data during home visits, ensuring anonymity and confidentiality. No missing data were recorded. Ethical approval was granted by the Research Ethics Committee of the University of West Attica before the study’s implementation.

The participants’ demographic characteristics were analyzed using descriptive statistics. Sampling adequacy (Kaiser-Meyer-Olkin, KMO = 0.62) was marginal but acceptable for exploratory purposes (Bartlett’s p < 0.01). An exploratory factor analysis (EFA) was employed to evaluate construct validity and identify potential underlying factors. Specifically, the varimax rotation method was used to examine correlations among the eight items. Varimax was chosen for interpretability, given low interfactor correlations (<0.3). In this case, the acceptable threshold for factor loadings was set at 0.4, and the eigenvalue threshold was set at one. Internal consistency was measured using Cronbach’s alpha for the factors derived from the EFA, with values above 0.7 indicating an acceptable level of internal reliability. Descriptive statistics were calculated for all items. Statistical analysis was performed using IBM Statistical Package for the Social Sciences Statistics for Windows, Version 29 (Released 2023; IBM Corp., Armonk, New York).

## Results

We identified two factors encompassing all eight variables of our questionnaire. The total variance explained by the questionnaire was 57.8%, indicating good construct validity. As shown in Table 1, the following two factors were identified: 1) satisfaction with the examination process and overall service quality (six variables) and 2) possible negative mental/physical effects due to the X-ray procedure (two variables). It is worth noting that the item concerning appointment availability exhibited moderate correlations with both extracted factors, consistent with its dual factor loadings (0.537 and -0.472). This supports its interpretation as a "bridge variable" linking service efficiency with emotional response. Specifically, the more satisfied patients were with appointment availability, the greater their overall satisfaction. However, this variable was also inversely associated with the second factor, suggesting that difficulties with appointment availability may increase anxiety or discomfort.

**Table 1:** Exploratory factor analysis for the eight items of the study questionnaire

Item	Factors	
	1	2
	Satisfaction with the examination process and overall service quality	Possible negative mental/physical effects due to the X-ray procedure
How satisfied are you with the information provided regarding the X-rays conducted through the home X-ray service?	0.810	-
Overall, how satisfied are you with the home X-ray service?	0.789	-
How satisfied are you with the cleanliness and adherence to hygiene standards during your examination?	0.752	-
How satisfied are you with the time it took to receive your results after your examination?	0.634	-
How satisfied are you with the care shown by the radiologic technologist during your examination?	0.568	-
How satisfied are you with the availability of your appointment for the examination?	0.537	-0.472
Did you experience anxiety during or after your examination?	-	0.871
Did you experience discomfort or pain during the examination?	-	0.855

The Cronbach’s alpha coefficients for the two factors derived from the EFA were greater than 0.7, indicating a high level of internal reliability. Specifically, the Cronbach’s alpha for the factor Satisfaction with the examination process and overall service quality was 0.74, while for the factor Possible negative mental/physical effects due to the X-ray procedure, it was 0.71. The KMO value of the questionnaire was 0.62, with a significant Bartlett’s test of sphericity (p < 0.01), demonstrating sample adequacy. The mean satisfaction scores in Table 2 indicate overall high satisfaction.

**Table 2:** Descriptive statistics for the eight items of the study questionnaire

Descriptive statistics	n	Minimum	Maximum	Mean	Standard deviation
Overall, how satisfied are you with the home X-ray service?	78	4	5	4.91	0.288
How satisfied are you with the information provided regarding the X-rays conducted through the home X-ray service?	78	3	5	4.79	0.466
How satisfied are you with the time it took to receive your results after your examination?	78	2	5	4.73	0.527
How satisfied are you with the availability of your appointment for the examination?	78	2	5	4.76	0.514
How satisfied are you with the cleanliness and adherence to hygiene standards during your examination?	78	4	5	4.90	0.305
How satisfied are you with the care shown by the radiologic technologist during your examination?	78	4	5	4.99	0.113
Did you experience discomfort or pain during the examination?	78	1	3	1.35	0.621
Did you experience anxiety during or after your examination?	78	1	3	1.31	0.517

A detailed demographic profile of the participants is presented in Table 3. The majority (74.4%) of participants were women. Most respondents (84.6%) were over 70 years old and retired. Furthermore, 91% were insured through EFKA, 64.1% used the service due to musculoskeletal conditions, and 93.5% reported an annual income of up to €30,000.

**Table 3:** Demographic characteristics of the participants

Characteristics	n (%)
Age (years)	
18-30	0 (0)
30-45	3 (3.8)
45-69	9 (11.5)
≥70	66 (84.6)
Gender	
Male	20 (25.6)
Female	58 (74.4)
Race	
Native	77 (98.7)
Foreigner	1 (1.3)
Educational level	
Upper 9 years	49 (62.8)
Lower 9 years	29 (37.2)
Insurance	
Public insurance provider	71 (91)
Private insurance	6 (7.7)
Uninsured	1 (1.3)
Annual income	
0-10,000€	22 (28.2)
10,001-20,000€	31 (39.7)
20,001-30,000€	20 (25.6)
30,001-40,000€	4 (5.1)
≥40,001€	1 (1.3)
Employment	
Full-time employment	9 (11.5)
Unemployed	3 (3.8)
Retired	66 (84.6)
Disease	
Noncommunicable disease	24 (30.8)
Contagious disease	4 (5.1)
Musculoskeletal disease	50 (64.1)

## Discussion

We conducted a reliability and validity study in a sample from the general population in Greece to develop a questionnaire measuring patient satisfaction with the home X-ray service. Our questionnaire has been shown to be both reliable and valid, and it was developed using a Likert scale [10], which is beneficial for conducting comparative studies and can be applied across different sociocultural contexts. It consists of eight questions that were simple and understandable for the participants.

Several questionnaires have been developed to measure user satisfaction with mobile X-ray units. Each questionnaire has a different structure, set of factors, and variables to meet the objectives of its creators. Moreover, every questionnaire has advantages and limitations, and each was designed for a specific population, while in some cases, they were completed by the patients’ relatives, while others were completed by caregivers [1,11,12]. In this context, we developed our own questionnaire to measure satisfaction with home X-ray services, completed by patients themselves.

The first of the two factors that emerged from the EFA was satisfaction with the examination process and overall service quality. Variables such as appointment availability, the time elapsed between the examination and receipt of results, adherence to hygiene standards, and the care provided by healthcare personnel during the examination were found to contribute significantly to satisfaction with the examination process [13].

Possible negative mental/physical effects due to the X-ray procedure were identified as the second factor. A deterrent for undergoing preventive and other examinations is often the fear and anxiety associated with hospital visits. It has been well-documented that, for the elderly population residing in nursing homes, a visit to the radiology department may constitute a distressing event, potentially leading to increased disorientation, delirium, or heightened anxiety [14,15].

The two-factor structure captures both procedural quality and psychological response dimensions, consistent with theoretical models such as SERVQUAL. The marginal KMO (0.62) suggests limited factorability but is acceptable for exploratory validation. Comparison with PSQ‑18 and SERVQUAL confirms conceptual overlap but highlights the novelty of a context‑specific tool for home radiography. Potential response bias exists due to the elderly sample and face‑to‑face administration. Results may not generalize beyond similar populations. Nevertheless, the findings provide preliminary validation evidence of a reliable, feasible instrument.

## Conclusions

The study of patient satisfaction is essential for evaluating the quality of healthcare services provided. Evaluating home X-ray services offers valuable insights for healthcare policy-making, supporting equitable access and the provision of high-quality care through the effective use of technology.

This study presents the initial validation of an eight-item questionnaire measuring patient satisfaction with home-based radiography services. The instrument demonstrated satisfactory construct validity and internal reliability. Future research will apply confirmatory factor analysis and validation in larger, more diverse, and cross-cultural samples.

## Appendices

**Table 4:** Questionnaire on patient satisfaction Responses are rated on a 5-point Likert scale: 1 = Not at all, 2 = A little, 3 = Moderately, 4 = Very, 5 = Completely

Questionnaire															
Please share your opinion about the home X-ray service by marking the appropriate field according to your answer. Your participation is voluntary. The questionnaire is anonymous, your responses are confidential, and they will not be disclosed. The estimated time to complete the questionnaire is approximately 5 minutes. We sincerely thank you in advance for your participation and the time you are willing to devote															
1. Overall, how satisfied are you with the home X-ray service?															
Not at all □	A little □		Moderately □						Very □			Completely □			
2. How satisfied are you with the information provided regarding the X-rays conducted through the home X-ray service?															
Not at all □	A little □		Moderately □						Very □			Completely □			
3. How satisfied are you with the time it took to receive your results after your examination?															
Not at all □	A little □		Moderately □						Very □			Completely □			
4. How satisfied are you with the availability of your appointment for the examination?															
Not at all □	A little □		Moderately □						Very □			Completely □			
5. How satisfied are you with the cleanliness and adherence to hygiene standards during your examination?															
Not at all □	A little □		Moderately □						Very □			Completely □			
6. How satisfied are you with the care shown by the radiologist technologist during your examination?															
Not at all □	A little □		Moderately □						Very □			Completely □			
7. Did you experience discomfort or pain during the examination?															
Not at all □	A little □		Moderately □						Very □			Completely □			
8. Did you experience anxiety during or after your examination?															
Not at all □		A little □		Moderately □					Very □			Completely □			
9. Age (years)															
18-30 □	30-45 □				45-69 □					Upper 70 years □					
10. Gender															
Male □	Female □											Other □			
11. Race															
Native □						Foreigner □									
12. Place of residence (municipality):															
13. Educational level															
Upper 9 years □												Lower 9 years □			
14. Insurance															
Public insurance provider □							Private insurance □					Uninsured □			
15. Annual income															
0-10,000 € □	10,001-20,000€ □					20,001-30,000€ □						30,001-40,000€ □			≥40,001€ □
16. Employment															
Full-time employment □											Unemployed □				Retired □
17. Please indicate the type of X-ray you received:															
18. Disease															
Noncommunicable disease □								Contagious disease □						Musculoskeletal disease □	
19. Questionnaire respondent															
Patient-respondent □													Companion/relative of the patient □		
As part of a doctoral dissertation, a study is being conducted on patient satisfaction with home X-ray services. The company AKTINOAPOIKONISI EXPRESS has granted the required authorization for the implementation of this study. The person responsible for the collection of the questionnaires is Panagiotis Bidikoudis. For any questions or clarifications, please contact us via email at: pbidikoudis@uniwa.gr															
